# Pulmonary Vein Stenosis—Evolving Surgical Management of a Challenging Disease

**DOI:** 10.3390/children8080631

**Published:** 2021-07-25

**Authors:** Eric N. Feins, Ryan Callahan, Christopher W. Baird

**Affiliations:** 1 Department of Cardiac Surgery, Boston Children’s Hospital and Harvard Medical School, Boston, MA 02115, USA; Christopher.Baird@cardio.chboston.org; 2 Department of Cardiology, Boston Children’s Hospital and Harvard Medical School, Boston, MA 02115, USA; Ryan.Callahan@cardio.chboston.org

**Keywords:** congenital heart defect, pulmonary vein stenosis, surgery, treatment

## Abstract

Pulmonary vein stenosis (PVS) is an extremely challenging clinical problem in congenital heart disease. It has traditionally required multimodal therapy given its complex underlying pathophysiology. As with other modalities, surgical therapy has undergone tremendous evolution since the 1950s. These evolving strategies have been based upon an improved understanding of the substrates that cause PVS and recurrent vein obstruction. More recent anatomic-based surgical strategies have focused on the pulmonary vein course, and how adjacent mediastinal structures can create a fulcrum effect on the pulmonary veins as they pass from the lung parenchyma to the left atrium. The consequent angulation of pulmonary veins creates altered wall shear stress and likely serves as a nidus for recurrent PVS. Encouraging early results suggest that eliminating pulmonary vein angulation and shortening/straightening the pulmonary vein course may prove effective in surgically managing PVS.

## 1. Introduction

Pulmonary vein stenosis (PVS) remains one of the most challenging problems in pediatric heart disease. It is well known that effective management of PVS requires an interdisciplinary team in order to implement multimodal therapy [1]. While medical and transcatheter therapies represent two cornerstones of treatment the surgical repair of PVS remains a critical component. Operative techniques to repair PVS have evolved over several decades, largely prompted by the ongoing challenges of recurrent stenosis and mortality. Multiple large reviews have documented mortality rates ranging from 46% to 58% in children with primary PVS [2,3,4,5]. Aggressive multimodal therapy including transcatheter/surgical intervention paired with tyrosine kinase receptor inhibition may improve patient survival to 77% but remains suboptimal [6]. Overall mortality may be better in patients undergoing the primary repair of total anomalous pulmonary venous connection (TAPVC) with contemporary three-year survival rates approximating 85% [7]. Nonetheless, post-repair pulmonary vein obstruction occurs in up to 15–21% of these patients [8,9] and remains an ongoing challenge with survival rates of only 58–73% [10,11]. This review will discuss the evolution of PVS surgery, with a particular emphasis on the principles underlying contemporary surgical techniques and newer repair strategies.

## 2. Historical Perspective

### 2.1. Early Surgical Techniques and Outcomes

Kawashima was one of the first to describe the repair of pulmonary vein stenosis in 1971 [12]. The surgery was performed three years prior on a 15-year-old boy with an atrial septal defect and suspected ventricular septal defect. Pulmonary vein disease was unexpectedly discovered upon opening the atrium. Obstruction by a membranous diaphragm was noted where the right upper pulmonary vein entered the left atrium with a 2 mm opening, and this was resected. Disease was also discovered on the left side, where there was localized thickening and stenosis at the junction of a common left pulmonary vein and the left atrium. This thickened tissue was incised to widen the stenotic area.

More complex repair techniques were subsequently developed to address more extensive disease and in younger patients. Bini and colleagues described several surgical techniques in a small series of eight patients ranging from newborn to four years of age [13]. These operations included excision of the stenotic region with direct re-implantation of the healthy pulmonary vein(s) into the left atrium, venoplasty akin to a Heinecke–Mikulicz pyloroplasty (i.e., longitudinal vein incision with transverse closure to widen the stenotic region), the use of prosthetic material (i.e., Gore-Tex^®^, Dacron^®^) to widen the vein-atrial junction, and the use of autologous atrial tissue to patch-enlarge the venous pathway. Autologous tissue reconstruction typically involved the left atrial appendage as an onlay patch onto the stenotic region of the left pulmonary veins, and the use of the interatrial septum to patch-enlarge the right-sided veins [14]. Unfortunately, early outcomes were quite sobering, particularly when the surgical repair was required early in life. Of the eight patients undergoing repair in Bini’s series, there were only five hospital survivors, all of whom developed rapid, progressive PVS recurrence and died within months of their operation [13]. So, while early surgical innovation aimed to tackle more complex pulmonary vein disease, PVS was still considered a “lethal lesion.”

### 2.2. TAPVC Repair Techniques and Outcomes

While an exhaustive account of TAPVC repair is beyond the scope of this review, for historical context it is worth noting that the surgical correction of TAPVC dates back to the 1950s. William H. Muller, Jr. first described the operative repair of “transposition of the pulmonary veins” in 1951 through a left thoracotomy, which involved a closed anastomosis between the left upper pulmonary vein and the left atrial appendage in a four-year-old girl [15]. Subsequent open anastomotic repair utilizing cardiopulmonary bypass was described by Denton Cooley in 1956 [16]. Reardon reported on the outcomes of TAPVC repair in 201 patients at the Texas Heart Institute spanning several decades from the 1950s to the 1980s. While survival improved dramatically with advances in surgical technique, cardiopulmonary bypass strategy, and perioperative care, younger patients, particularly those requiring surgery in the newborn period, remained at particularly high risk of mortality following TAPVC repair. In children undergoing repair at less than one month of age, mortality was 57%, and survivors were still subject to the risk of anastomotic stenosis [17].

Trans-atrial repair of TAPVC also evolved from Denton Cooley’s original technique. The goal of the trans-atrial approach to TAPVC repair was to maintain the heart in the orthotopic position when setting up and fashioning the vein-atrium anastomosis so as to avoid any distortion that might predispose to stenosis. Doty and colleagues endorsed this strategy and reported on improved outcomes (*n* = 20 patients) after the adoption of the trans-atrial approach in 1976, with a reduction in mortality from 57% to 8% [18]. Their findings emphasized the exquisite sensitivity to geometric distortion of the pulmonary vein-left atrial anastomosis, with subtle geometric changes causing kinking/obstruction, in addition to turbulence with subsequent intimal hyperplasia and recurrent stenosis.

## 3. Evolving Surgical Strategy—The Sutureless Repair

### 3.1. The Conceptual Basis and Early Development

**Figure 1:**
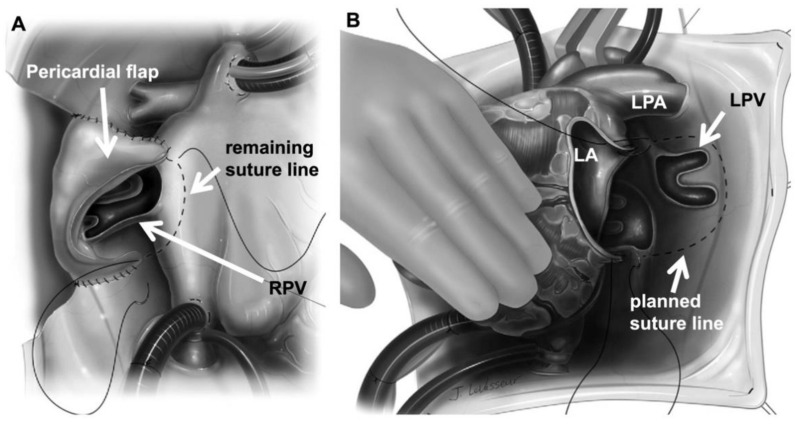
Sutureless repair. (**A**) Right pulmonary vein repair. Veins are opened widely and diseased tissue is resected. Pericardial flap is folded down and sewn to left atrium leaving cut edge of veins without sutures; (**B**) Left pulmonary vein repair. Heart is retracted rightward. Left pulmonary veins are widely opened and left atrial appendage is sewn to pericardium outside/around veins. (RPV = right pulmonary veins; LA = left atrium; LPV = left pulmonary veins; LPA = left pulmonary artery; Dashed lines = planned suture line for left atrial-pericardial anastomosis). Adapted from Viola, N.; Calderone, C.A. Surgical repair of post-repair pulmonary vein stenosis using “sutureless” techniques. Op Tech Thorac Cardiovasc Surg 2011, 15, 112–121.

Given the overall sobering outcomes of primary PVS repair, as well as the significant problem of post-TAPVC repair pulmonary vein obstruction, surgical techniques continued to evolve. In particular, “sutureless” repair techniques were developed in the 1990s by multiple groups. Lacour-Gayet and colleagues in Paris first described the sutureless repair in 1996 specifically to address post-TAPVC repair pulmonary vein obstruction in a two-and-a-half-year-old child [19]. Like others, their group had experienced suboptimal outcomes with conventional techniques (i.e., endarterectomy +/− patch enlargement) to address post-TAPVC repair pulmonary vein obstruction. In a review of 16 patients undergoing surgery for post-TAPVC repair vein obstruction, there was a 27% mortality rate [20]. In part inspired by the Senning operation for physiologic correction of D-transposition of the great arteries, the sutureless technique involved the use of an in situ vascularized pericardial flap which was sewn directly to the atrial wall thereby avoiding manipulation/suturing directly to the pulmonary vein tissue (Figure 1A). The virtue of this technique was based upon the concern that manipulation/suturing on vein tissue served as a nidus for inflammation, scar formation, and recurrent stenosis. Moreover, suturing the atrium to the pericardium was felt to allow for a more aggressive resection of diseased vein tissue since the surgeon would not have to rely on unresected vein tissue onto which to sew.

The area of diseased pulmonary vein on the right side was unroofed, or ideally completely resected, and by sewing the in situ autologous pericardial flap to the atrium, a widely patulous pathway was created for the right-sided pulmonary venous blood to return to the left atrium. Najm and colleagues in Toronto described a similar approach of using an autologous pericardial flap to open up the pulmonary vein-atrial connection while avoiding a direct anastomosis to vein tissue in two patients [21]. In both reports, the pericardial flap was only applied on the right side. Left-sided PVS was addressed through the left atrium by incising longitudinally out into the left pulmonary veins getting outside of the heart. Instead of using an in situ pericardial flap to contain the left pulmonary vein flow, pericardial adhesions from the prior TAPVC repair were relied upon to direct pulmonary vein-to-atrial blood flow without hemorrhage.

In their review of acquired PVS management, Spray and Bridges described an alternative for the left-sided pulmonary veins which did not rely upon previous pericardial adhesions. This involved incising into the left atrial appendage and out into the left-sided veins, carrying this incision out to the pericardial reflection. The opened left atrial appendage was then sewn down as a flap directly to the posterior pericardial reflection [22]. Analogous to the right side, described by Lacour-Gayet and Najm, this created a widely patulous pulmonary venous pathway into the left atrium without a direct pulmonary vein anastomosis. Because post-operative adhesions were not relied on to contain the left-sided pulmonary venous flow this maneuver enabled sutureless repair to be applied for primary PVS, not just post-repair PVS (Figure 1B).

### 3.2. Sutureless Repair Outcomes and Scope of Use

Outcomes for the treatment of post-TAPVC repair PVS with the sutureless technique have generally been favorable when compared with older techniques. In a review of surgery for PVS after TAPVC repair ten years after introducing the technique, Lacour-Gayet reported an improvement in freedom from mortality or recurrent PVS from 65% to 90% [23]. Devaney and colleagues also noted improved disease-free survival in a study of 22 patients when using a sutureless pericardial marsupialization for PVS following TAPVC repair [24].

Given these encouraging results, some groups have expanded their use of the sutureless repair. Calderone and colleagues describe extending the sutureless repair to all forms of PVS, including primary repair of TAPVC [25,26]. Aside from the avoidance of direct suturing to pulmonary vein tissue (noted above), the sutureless repair is thought to simplify the anastomosis. Since the suture lines are atrio-pericardial, the anastomosis is felt to be less prone to distortion in the way that direct vein-atrium connections might be, particularly when the geometry/orientation of the pulmonary vein confluence would make direct anastomosis to the back of the left atrium challenging. In a study of 22 patients, Calderone and colleagues compared sutureless and conventional repair strategies for the primary correction of mixed-type TAPVC and found a non-significant trend toward improved survival and freedom from re-intervention in the sutureless repair group [27].

Importantly, outcomes for sutureless repair of primary PVS have been less encouraging. Kanter and colleagues found no significant difference in freedom from recurrent PVS or death when comparing 52 patients who received either a standard repair or a sutureless repair [28,29]. Similarly, Shi and colleagues saw no difference in outcomes among surgical techniques (including sutureless repairs) in 18 patients with primary PVS [27]. In a multicenter study looking at primary PVS in 30 patients, Kalfa and colleagues found no association between the use of a sutureless technique and improved outcomes [30]. Calderone’s group also found that mortality and restenosis rates remained high in a review of 23 patients despite the adoption of a sutureless repair strategy for primary PVS patients [31]. These variable outcomes largely speak to the complex, multifactorial pathophysiology underlying PVS. Sutureless repair techniques may aim to avoid direct manipulation and suturing on the pulmonary veins, however, other factors are at play that drive the over-activity of myofibroblast-like cells in the pulmonary vein subendothelium leading to PVS recurrence [32,33].

## 4. New PVS Repair Concepts and Strategies—The Fulcrum Effect and the Anatomic-Based Repair

### 4.1. External Anatomy—The Impact on Pulmonary Veins

While much of the focus on PVS has been on the pulmonary veins themselves, little attention has been paid to the surrounding intrathoracic structures that can impact the pulmonary vein course. At our institution, we have increasingly recognized, through pre-operative imaging, and intraoperatively, a fulcrum effect on the pulmonary veins of our PVS patients. We have observed that as the pulmonary veins pass from the lung parenchyma they must pivot over/pass neighboring mediastinal structures and across the pericardial reflection before reaching the back of the left atrium more medially. This creates a long, angulated, and tortuous pulmonary vein course from the lung parenchyma to the heart. It is well known that vessel angulation leads to variations in wall shear stress, a phenomenon well described by Han [34]. Importantly, changes to vessel wall shear stress contribute to vascular remodeling, intimal hyperplasia, and stenosis [35].

**Figure 2:**
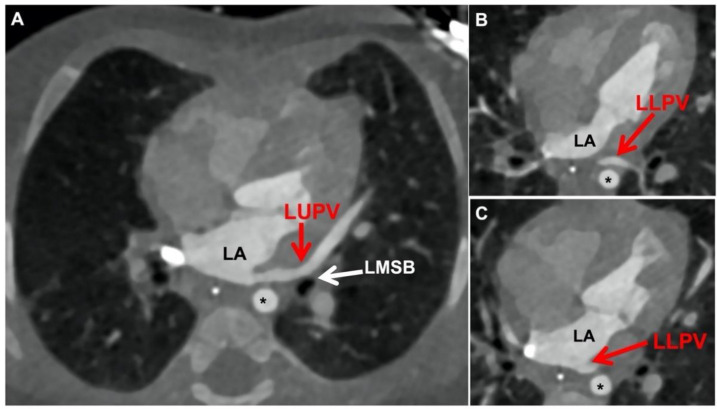
Left pulmonary veins (CT images). (**A**) Left upper pulmonary vein takes long, angulated course as it passes from lung parenchyma over left mainstem bronchus, across pericardial reflection into left atrium medially; (**B**) Left lower pulmonary vein courses anteriorly over the descending thoracic aorta taking an angulated course; (**C**) Left lower pulmonary vein crossing the pericardial reflection and entering far medially into left atrium. (LA = left atrium; LUPV = left upper pulmonary vein; LMSB = left mainstem bronchus; LLPV = left lower pulmonary vein; Asterisk = descending thoracic aorta).

The specific mediastinal structures that impact the pulmonary vein course vary with location. The left upper pulmonary vein must pass anteriorly over the left mainstem bronchus and then pass across the pericardial reflection before entering the left atrium more medially and posteriorly. Enlargement of the bronchus in the setting of chronic lung disease with elevated airway pressures can exacerbate pulmonary vein angulation (Figure 2A). The left lower pulmonary vein must pass anteriorly over the descending thoracic aorta and then cross the pericardial reflection before reaching the left atrium more medially (Figure 2B,C). In their analysis of left-sided PVS in single-ventricle patients, Kotani and colleagues described this relationship of the left pulmonary veins and the descending thoracic aorta [36]. Using MRI data they demonstrated that an anterolaterally displaced descending thoracic aorta in combination with cardiomegaly predisposed single-ventricle patients to left lower pulmonary vein stenosis. We have anecdotally observed that left lower lobe atelectasis is a common finding in PVS patients who have chronic lung disease, and this can pull the left lower vein more posteriorly, thus exacerbating this vessel angulation.

**Figure 3:**
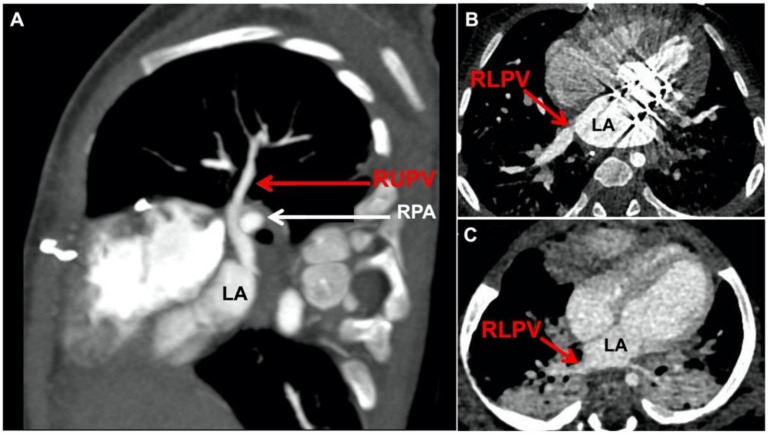
Right pulmonary veins (CT images). (**A**) Right upper pulmonary vein crosses over right pulmonary artery causing angulation as it passes across pericardial reflection into left atrium; (**B**) Right lower pulmonary vein stenosis involves more posteriorly directed pulmonary vein that angulates when crossing pericardial reflection; (**C**) Right lower pulmonary vein without stenosis has straight, less angulated course into left atrium. (RUPV = right upper pulmonary vein; RPA = right pulmonary artery; RLPV = right lower pulmonary vein; LA = left atrium).

The right upper pulmonary vein angulates over the right pulmonary artery and then must pass across the pericardial reflection before entering the left atrium more medially (Figure 3A). Importantly, when pulmonary hypertension exists, a common condition with PVS, the pulmonary arteries are enlarged and hypertensive, which accentuates their impact on the pulmonary vein course. The right lower pulmonary vein is the least commonly involved vessel in PVS, a finding noted in an analysis by Callahan and colleagues of the disease patterns and outcomes for the specific pulmonary veins [37]. When right lower PVS does occur there is angulation as the vein passes across the pericardial reflection and into the left atrium. This is typically seen with a more posteriorly directed right lower pulmonary vein (Figure 3B), in contrast to the more typical orientation that has a straight pathway to the left atrium (Figure 3C).

### 4.2. The Anatomic-Based Repair

Given the recognition of the long, angulated course that the pulmonary veins can take as they pass from the lung parenchyma to the left atrium, we now place greater emphasis on the extrinsic anatomy when repairing PVS of all forms [38]. The goal of the anatomic-based PVS repair strategy is to remove the fulcrum effect that adjacent mediastinal structures and the pericardial reflection can have on the pulmonary veins, and create a shorter and straighter course of the veins into the left atrium.

#### 4.2.1. Pre-Operative Evaluation

Pre-operatively, this involves having a clear understanding of both the extent/location of the pulmonary vein disease as well as the actual course of the veins in the mediastinum. Cardiac catheterization remains the gold standard for assessing pulmonary vein disease severity and includes hemodynamics, angiography, pressure assessment, intravascular ultrasound, and balloon compliance testing. In addition, we are increasingly using pre-operative computed tomography (CT) for surgical planning in order to get a more global sense of the pulmonary veins, the adjacent mediastinal structures, and the degree of vessel angulation/tortuosity (Figure 2 and Figure 3).

#### 4.2.2. Operative Technique

The primary components of the anatomic-based PVS repair strategy are:

1. Thorough takedown of the pericardial reflection and aggressive mobilization of the pulmonary veins as they enter the pericardial space, in order to eliminate any angulation as the veins cross the pericardium;
2. Resection of any stenotic or thickened pulmonary vein tissue;
3. Lateralizing and enlargement of the pulmonary vein-left atrial junction, such that the course of the pulmonary veins into the left atrium is shorter, less angulated, and therefore less prone to disturbed flow.

**Figure 4:**
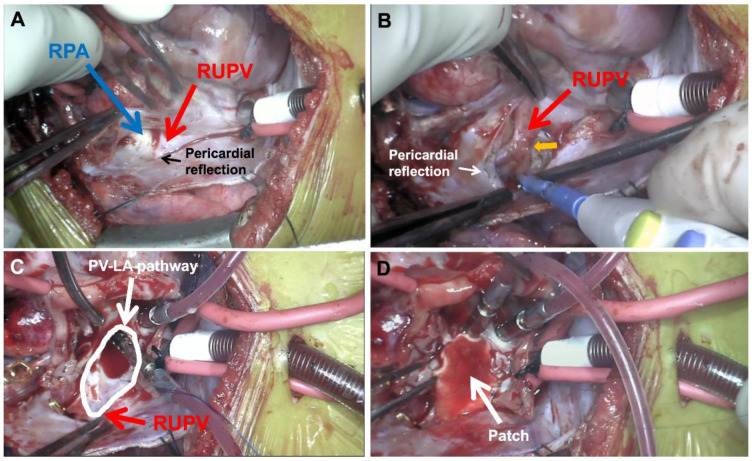
Right pulmonary vein repair. (**A**) Right-sided pulmonary veins crossing pericardial reflection before entering left atrium more medially; (**B**) Complete mobilization of pericardial reflection and right pulmonary veins with stenotic region denoted (yellow arrow); (**C**) Right pulmonary veins widely opened up and diseased tissue resected creating patulous pulmonary vein-left atrial pathway (white outline); (**D**) Patch augmentation moves pulmonary vein-left atrial connection out laterally to shorten/straighten pulmonary vein course. (RUPV = right upper pulmonary vein; RPA = right pulmonary artery; PV-LA = pulmonary vein-left atrium).

Figure 4 depicts operative repair of right-sided pulmonary vein disease. Figure 4A demonstrates the right pulmonary veins prior to repair. They course across the pericardial reflection, as depicted in the figure, and then enter the left atrium quite medially. The first operative step involves complete takedown of the pericardial reflection, during which the pulmonary veins are freed up completely out into the lung parenchyma (Figure 4B). Importantly, the region of stenosis typically correlates with where the veins crossed the pericardial reflection, as denoted in the figure (yellow arrow). The veins are opened up in this region to create a wide, patulous pathway from the lung parenchyma into the left atrium, depicted by the white outline in Figure 4C. The pulmonary vein-left atrial connection is then patched, which enlarges the pathway and helps move the vein-atrial connection more laterally (Figure 4D). Various patch materials have been used for this part of the repair. A thin-patch pulmonary homograft is often used given its compliant tissue characteristics. Importantly, we have a preference for using a decellularized homograft given its decreased immunogenicity, an important consideration for a patient population that may require lung transplantation [39]. Alternatively, we have used autologous atrial flaps to lateralize and augment the pulmonary vein-left atrial pathway, thereby using only native tissue on the pulmonary veins themselves.

**Figure 5:**
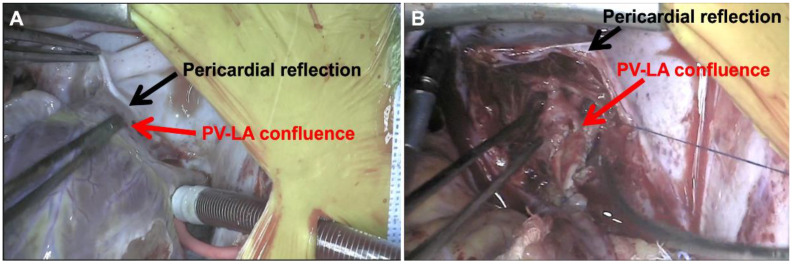
Left pulmonary vein repair. (**A**) Pre-repair—Left pulmonary veins cross pericardial reflection and enter back of left atrium more medially; (**B**) Post-repair—Pericardial reflection has been taken down and PV-LA confluence is straighter/lateralized. (PV-LA = pulmonary vein-left atrium).

Figure 5A demonstrates the medial entrance of the left pulmonary veins into the left atrium as they cross the pericardial reflection. Akin to the right-sided veins, the pericardial reflection is aggressively taken down and the veins are mobilized out into the lung parenchyma. The veins are opened out beyond the stenosed region and diseased tissue is resected. The venotomy is extended into the base of the left atrial appendage and the vein-atrial connection is patch-augmented to lateralize the vein-atrial confluence. Figure 5B demonstrates the repaired veins, where the left atrium has been effectively lateralized by the patch, and the pericardium is well away from the veins, thereby alleviating the fulcrum effect.

**Figure 6:**
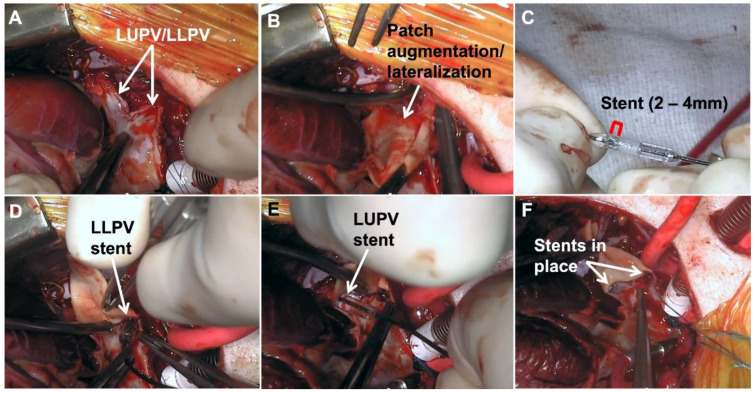
Intraoperative stent placement. (**A**) Hypoplastic left upper and lower pulmonary veins; (**B**) Patch enlargement and lateralization of vein-atrial connection; (**C**) Stent trimmed to ~3 mm and predilated over balloon; (**D**) Placement of left lower pulmonary vein stent; (**E**) Placement of left upper pulmonary vein stent; (**F**) Final position of stents in left veins to maintain luminal caliber and prevent compression. (LUPV = left upper pulmonary vein; LLPV = left lower pulmonary vein).

In select cases, we have opted to place a short stent at the pulmonary vein-left atrial connection as part of the repair. This is typically reserved for cases with severely hypoplastic or atretic pulmonary veins (i.e., less than 2 mm in luminal diameter), where there is concern about the pulmonary vein-left atrial connection getting compressed and narrowing down despite patch enlargement. To date, we have place stents in six patients at the time of PVS repair. Figure 6A shows small left upper and lower pulmonary veins despite opening out into them with resection of diseased tissue. A note is made of the mobilization and takedown of the pericardial reflection. After patch augmenting and lateralizing the vein-atrial connection (Figure 6B), a stent is trimmed to ~3 mm and pre-dilated with particular attention paid to not over-dilate the stent (Figure 6C). Separate stents are placed at the ostia of the left lower (Figure 6D) and left upper pulmonary veins (Figure 6E). These stents are meant to maintain the luminal size of the reconstructed veins (Figure 6F). A critical operative step is to secure the stents in place with multiple 7-0 polypropylene sutures to prevent dislodgement. Importantly, the stents act as a substrate for future transcatheter dilation in these cases of hypoplastic pulmonary veins.

#### 4.2.3. Post-Operative Evaluation and Management

**Figure 7:**
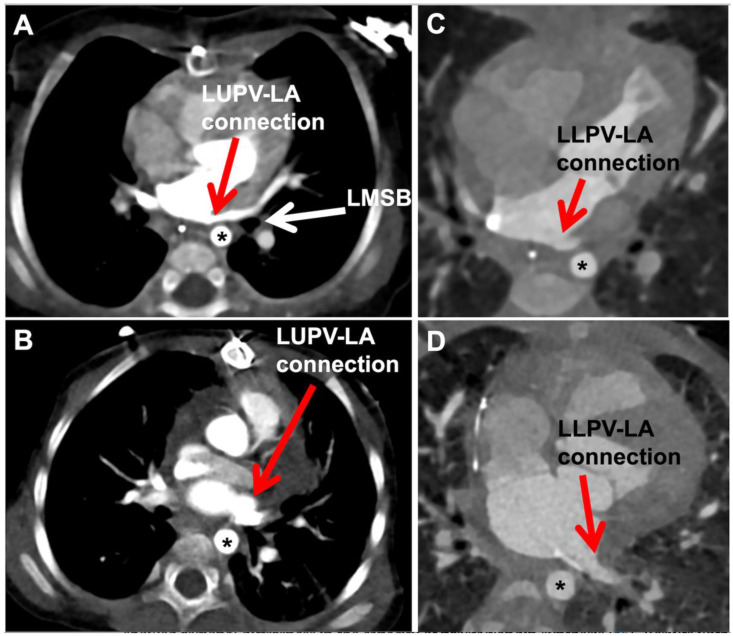
Pre- and post-repair pulmonary vein CT imaging. (**A**) Pre-operative left upper pulmonary vein taking long course over left bronchus before entering medially into left atrium; (**B**) Post-repair left upper vein course is shorter and more lateral—minimizing angulation; (**C**) Pre-operative left lower vein entering left atrium very medially after coursing over descending aorta; (**D**) Post-repair left lower vein takes shorter/straighter course as it enters atrium more laterally. (LUPV-LA = left upper pulmonary vein-left atrium; LMSB = left mainstem bronchus; LLPV-LA = left lower pulmonary vein-left atrium; Asterisk = descending thoracic aorta).

Patients are maintained on therapeutic anticoagulation with unfractionated heparin that is transitioned to low-molecular-weight heparin, along with aspirin. Standard post-operative surveillance involves echocardiography paired with nuclear lung perfusion scans. These studies are performed monthly for three months, and then less frequently if there is no evidence of PVS recurrence. Post-operative axial imaging is being performed more frequently to assess the anatomic result of this repair strategy, although there is not currently a formalized protocol for the timing/frequency of post-operative axial imaging. We have observed effective lateralization of the pulmonary vein-atrial connection to shorten the course of the veins as they pass into the left atrium. Figure 7 demonstrates this effect on the left upper and lower veins. Pre-operatively, the left upper vein passes over the bronchus and across the pericardial reflection before entering the left atrium medial to the descending aorta (Figure 7A). After repair, the left upper vein takes a shorter course, away from the airway, and enters the left atrium more laterally (Figure 7B). Similarly, pre-operative imaging of the left lower vein shows its more medial entry into the left atrium after coursing over the descending aorta (Figure 7C). Following repair, the left lower vein now enters more laterally and has a shorter/straighter course into the left atrium without pivoting over the aorta (Figure 7D).

Post-operative chemotherapy is reserved for patients with intraluminal PVS (presence of fibromyxoid proliferation) confirmed by a pre-operative biopsy or by angiography and intravascular ultrasound. Excluded patients are those with single-vessel PVS and those with pulmonary venous obstruction isolated to a surgical anastomosis or confluence without individual vessel involvement as determined by cardiac catheterization. Eligible patients are started on imatinib for targeted anti-proliferative therapy 7–10 days after surgery to allow for adequate wound healing.

#### 4.2.4. Preliminary Outcomes

This newer anatomic-focused approach has yielded encouraging results to date. While only implemented in the last several years, we have observed an improvement in mortality when compared to more conventional PVS repair techniques, with a two-year survival of 82.1% versus 61.8% for conventional repair (*p* = 0.03) [40]. Importantly, this mortality benefit was observed after controlling for potential confounding factors, including age, number of vessels involved, surgical era, and use of adjuvant chemotherapy. Nonetheless, ongoing surveillance is crucial to better understand the longer-term durability of this repair strategy. We have not observed a significant change in the re-intervention rate, however, re-intervention, especially transcatheter, should not be considered a “failure”. PVS is a challenging and chronic disease, and the expectations should be that despite evolving surgical techniques, re-intervention is a likely and even expected management strategy.

## 5. Conclusions

The multimodal treatment of PVS has evolved tremendously over the past several decades, with advances in medical, transcatheter, and operative therapies. Surgical repair of PVS remains a central component of treatment, and techniques of repair have developed with our increasing understanding of the underlying pathophysiology of PVS. While conventional venoplasty techniques initially rendered poor outcomes, sutureless techniques sought to eliminate the risk of geometric distortion and scar formation related to direct pulmonary vein manipulation. The sutureless techniques proved to be effective in certain circumstances, however, they have not been the panacea for PVS. The more recent focus on the pulmonary vein course, angulation, and the fulcrum effect has expanded our conception of how best to correct the anatomic substrates leading to PVS. Newer anatomic-based techniques aimed at eliminating the fulcrum/angulation have rendered promising outcomes thus far. As with every novel therapy for PVS, it will be critical to follow these patients closely to understand whether this new approach has a lasting impact. Importantly, given the heterogeneity of PVS, it is unlikely that one operative strategy will work for all forms of the disease. In that regard, continued surgical evolution will be important to further enhance our operative “toolkit” for the treatment of this challenging disease.

## Data Availability

Not applicable.
